# Authentication and characterisation of a new oesophageal adenocarcinoma cell line: MFD-1

**DOI:** 10.1038/srep32417

**Published:** 2016-09-07

**Authors:** Edwin Garcia, Annette Hayden, Charles Birts, Edward Britton, Andrew Cowie, Karen Pickard, Massimiliano Mellone, Clarisa Choh, Mathieu Derouet, Patrick Duriez, Fergus Noble, Michael J. White, John N. Primrose, Jonathan C. Strefford, Matthew Rose-Zerilli, Gareth J. Thomas, Yeng Ang, Andrew D. Sharrocks, Rebecca C. Fitzgerald, Timothy J. Underwood, OCCAMS consortium, Shona MacRae, Nicola Grehan, Zarah Abdullahi, Rachel de la Rue, Ayesha Noorani, Rachael Fels Elliott, Nadeera de Silva, Jan Bornschein, Maria O’Donovan, Gianmarco Contino, Tsun-Po Yang, Hamza Chettouh, Jason Crawte, Barbara Nutzinger, Paul A. W. Edwards, Laura Smith, Ahmad Miremadi, Shalini Malhotra, Alison Cluroe, Richard Hardwick, Jim Davies, Hugo Ford, David Gilligan, Peter Safranek, Andy Hindmarsh, Vijayendran Sujendran, Nick Carroll, Richard Turkington, Stephen J. Hayes, Yeng Ang, Shaun R. Preston, Sarah Oakes, Izhar Bagwan, Vicki Save, Richard J. E. Skipworth, Ted R. Hupp, J. Robert O’Neill, Olga Tucker, Philippe Taniere, Jack Owsley, Charles Crichton, Christian Schusterreiter, Hugh Barr, Neil Shepherd, Oliver Old, Jesper Lagergren, James Gossage, Andrew Davies, Fuju Chang, Janine Zylstra, Grant Sanders, Richard Berrisford, Catherine Harden, David Bunting, Mike Lewis, Ed Cheong, Bhaskar Kumar, Simon L. Parsons, Irshad Soomro, Philip Kaye, John Saunders, Laurence Lovat, Rehan Haidry, Victor Eneh, Laszlo Igali, Ian Welch, Michael Scott, Shamila Sothi, Sari Suortamo, Suzy Lishman, Duncan Beardsmore, Charlotte Anderson, Mike L. Smith, Maria Secrier, Matthew D. Eldridge, Lawrence Bower, Achilleas Achilleos, Andy G. Lynch, Simon Tavare

**Affiliations:** 1 Faculty of Medicine, University of Southampton, Southampton General Hospital, Mailpoint 801, South Academic Block, Tremona Road, Southampton, SO16 6YD, United Kingdom; 2 Faculty of Biology, Medicine and Health, Oxford Road, University of Manchester, Manchester, M13 9PT, UK; 3 MRC Cancer Unit, University of Cambridge, Hutchison/MRC Research Centre, Box 197, Cambridge Biomedical Campus, Cambridge, CB2 0XZ United Kingdom; 4 Department of Histopathology, Addenbrooke’s Hospital, Cambridge, UK.; 5 Oesophago-Gastric Unit, Addenbrooke’s Hospital, Cambridge, UK.; 6 Oxford ComLab, University of Oxford, UK.; 7 Cambridge University Hospitals NHS Foundation Trust, Cambridge, UK.; 8 Centre for Cancer Research and Cell Biology, Queen’s University Belfast, Northern Ireland, UK.; 9 Salford Royal NHS Foundation Trust, Salford, UK.; 10 Wigan and Leigh NHS Foundation Trust, Wigan, Manchester, UK.; 11 Royal Surrey County Hospital NHS Foundation Trust, Guildford, UK.; 12 Edinburgh Royal Infirmary, Edinburgh, UK.; 13 University Hospitals Birmingham NHS Foundation Trust, Birmingham, UK.; 14 University Hospital Southampton NHS Foundation Trust, Southampton, UK.; 15 Faculty of Medical and Human Sciences, University of Manchester, UK.; 16 Department of Computer Science, University of Oxford, UK.; 17 Gloucester Royal Hospital, Gloucester, UK.; 18 St Sharrocks’s Hospital, London, UK.; 19 Plymouth Hospitals NHS Trust, Plymouth, UK.; 20 Norfolk and Norwich University Hospital NHS Foundation Trust, Norwich, UK.; 21 Nottingham University Hospitals NHS Trust, Nottingham, UK.; 22 University College London, London, UK.; 23 Norfolk and Waveney Cellular Pathology Network, Norwich, UK.; 24 Wythenshawe Hospital, Manchester, UK.; 25 Edinburgh University, Edinburgh, UK.; 26 King’s College London, London, UK.; 27 Karolinska Institutet, Stockholm, Sweden.; 28 University Hospitals Coventry and Warwickshire NHS, Trust, Coventry, UK.; 29 Peterborough Hospitals NHS Trust, Peterborough City Hospital, Peterborough, UK.; 30 Royal Stoke University Hospital, UHNM NHS Trust, UK.; 31 Institute of cancer and genomic sciences, University of Birmingham.; 32 GI science centre, University of Manchester, UK.; 33 Cancer Research UK Cambridge Institute, University of Cambridge, Cambridge, UK.

## Abstract

New biological tools are required to understand the functional significance of genetic events revealed by whole genome sequencing (WGS) studies in oesophageal adenocarcinoma (OAC). The MFD-1 cell line was isolated from a 55-year-old male with OAC without recombinant-DNA transformation. Somatic genetic variations from MFD-1, tumour, normal oesophagus, and leucocytes were analysed with SNP6. WGS was performed in tumour and leucocytes. RNAseq was performed in MFD-1, and two classic OAC cell lines FLO1 and OE33. Transposase-accessible chromatin sequencing (ATAC-seq) was performed in MFD-1, OE33, and non-neoplastic HET1A cells. Functional studies were performed. MFD-1 had a high SNP genotype concordance with matched germline/tumour. Parental tumour and MFD-1 carried four somatically acquired mutations in three recurrent mutated genes in OAC: *TP53*, *ABCB1* and *SEMA5A*, not present in FLO-1 or OE33. MFD-1 displayed high expression of epithelial and glandular markers and a unique fingerprint of open chromatin. MFD-1 was tumorigenic in SCID mouse and proliferative and invasive in 3D cultures. The clinical utility of whole genome sequencing projects will be delivered using accurate model systems to develop molecular-phenotype therapeutics. We have described the first such system to arise from the oesophageal International Cancer Genome Consortium project.

Oesophageal cancer is hard to treat. Patients typically present with late-stage disease and respond poorly to conventional anti-cancer therapies^1^. In the case of oesophageal adenocarcinoma (OAC) rates of diagnosis and death run roughly parallel and there have been few improvements in outcomes in the last 30 years^2^.

Recent efforts have been directed towards understanding the genetic landscape of OAC with a view to the identification of potentially targetable driver mutations. These studies have revealed the complexity of the genomic aberrations in OAC. The progression from Barrett’s Oesophagus (BE), the only known precursor lesion from OAC, to invasive cancer is characterised by early chromosomal instability, probably due to p53 loss, often including genome doubling and a high frequency of chromothripsis events resulting in considerable genetic diversity followed by a later acquisition of driver mutations at sub-clonal frequencies^3^,^4^,^5^. By tracking the genomic evolution of OAC through neoadjuvant chemotherapy (NAC) treatment and surgical resection, Murugaesu *et al.* have further revealed an association between high intratumour heterogeneity and poor response to NAC^6^. Such complexity makes the identification of tractable therapeutic targets difficult and necessitates the development of *in-vitro* models that represent the mutational burden of the primary tumour.

Unfortunately there is a paucity of such tools with which to study OAC. This lack of suitable preclinical models is frustrating efforts to understand both the basic biology of the transition from BE to OAC and the development of novel therapies for established cancer^7^. BE is confined to humans and some primates^8^ but the logistics of performing research in large mammals and the anatomical interspecies differences in the oesophago-gastric junction between humans and rodents has limited representative animal modelling^9^,^10^. Research on OAC is therefore dependant on a very small set of established cell lines, with little known about the genomic landscape of the tumour of origin or how representative the cell lines are of the genetic aberrations within the primary cancer. In a world-wide effort to validate the authenticity of 13 of the 14 OAC cell lines available in 2010, Boonstra *et al.* found that 3 frequently used lines originated from other tissues (lung cancer, colorectal cancer, gastric cancer)^11^. Over 100 publications, three National Institute of Health grants and 11 US patents have been founded on these cell lines. These concerns are not confined to oesophageal cancer research. When the National Cancer Institute’s Developmental Therapeutics Program (DTP) panel of 60 cancer cell lines derived from nine different tumour types was assessed for relevance in the study of clinical multidrug resistance (MDR) mechanisms it was found that the cell lines bore more resemblance to each other, regardless of the tissue of origin, than to the clinical samples they were supposed to model^12^. In OAC, even for the verified cell lines that are currently used, no data is available for the matched primary tissue.

In an attempt to address these weaknesses a number of groups have used alternative strategies for the establishment of *in-vitro* models in OAC, such as the immortalisation and malignant transformation of Barrett’s metaplastic epithelial cells by disrupting key signalling pathways and telomerase overexpression^13^. New, well-described and authenticated, cell lines derived from OAC without any *in-vitro* modification are valuable tools, such as the recently introduced OANC1^14^,^15^. But these cell lines may or may not be representative of the mutational profile of cells driving the malignancy *in vivo*^16^.

Using complementary genome wide analysis techniques including whole genome sequencing and genotyping of tumour biopsy and matched normal tissue from the oesophagus and peripheral blood we describe the establishment of a new model system in OAC (MFD-1) that retains the mutational profile of the primary tumour, including the identification of novel mutations in recognised cancer associated genes in OAC. Using a whole-transcriptome approach we compare and contrast the expression of recognised OAC associated genes in MFD-1 with established cell lines commonly used to study OAC. We show that MFD-1 is a highly representative model of OAC, it is stable over time and retains the ability to form tumours in xenograft models that respond to microenvironmental stimuli.

## Materials and Methods

### Ethics

The study was approved by the Southampton and South West Hampshire Research Ethics Committee (Oesophagus: molecular, cellular and immunological assessment. LREC number: 09/H0504/66). The methods were carried out in accordance with the approved guidelines and written informed consent was obtained prior to the study.

### Patient clinicopathological chacteristics

The MFD-1 cell line was derived from a 55 year-old white male with locally invasive adenocarcinoma of the oesophagus clinically and radiologically (CT, PET & EUS) staged as T3N1M0^17^. The patient was treated with 3 cycles of neoadjuvant chemotherapy (Epirubicin, Oxaliplatin and Capecitabine) prior to oesophagectomy. Histopathological analysis revealed a ypT4N3(15/26)M0 tumour with complete microscopic surgical resection (R0). The Mandard tumour regression grade (TRG)^1^,^18^ score was 5. Barrett’s oesophagus was not observed in the resection specimen. The patient died seven months after surgery from recurrent disease.

### Isolation of Tumour cells and establishment of the OAC cell line

The specimen was transferred directly from the operating theatre to the research laboratory in complete DMEM media (10% FBS, Penicillin streptomycin) on ice, washed with 10 ml of phosphate buffered saline 3 times and minced with a scalpel to reach approximately 3 mm^3^ pieces that were subsequently digested with trypsin-EDTA (Sigma, Missouri-USA) at 37 °C in a 5% humidified atmosphere for 30 minutes. Undigested tissues and debris were removed by sedimentation and the clear supernatant was collected and spun at 600 g for 5 minutes. Cell pellets were cultured in complete DMEM under standard cell culture incubation conditions. The cell line has been deposited in the European Collection of Authenticated Cell Cultures (ECACC), a standard resource for the academic research community. Individual researchers wishing to verify the findings in this study can also approach the corresponding author directly to obtain frozen cell stocks.

### SNP6 array genotyping

Genotyping of genomic DNA from peripheral blood mononuclear cells (PBMC), tumour and adjacent normal squamous oesophageal epithelium, and two early passages of the MFD-1 cell line (P1 & P4) were performed according to manufacturer recommendations (Affymetrix, California USA)^19^. The SNP6 array contained 906,000 probes for the genotyping of SNPs and 946,000 probes for the genotyping of non-polymorphic copy number. Affymetrix CEL files were analysed using the tool PICNIC^20^ (predicting absolute allele copy number variation with microarray cancer data). Data are deposited with ArrayExpress (Accession number E-MTAB-4590).

### Whole genome sequencing

Whole genome sequencing (WGS) from snap-frozen oesophageal tumour tissue and germline nucleic acids isolated from peripheral blood mononuclear cells (PBMC) was performed as part of the International Cancer Genome Consortium project and OCCAMS consortium as previously described^4^,^21^. Filtered read sequences were mapped to the human reference genome (GRCh37) using Burrows-Wheeler Alignment (BWA) (Accession number E-MTAB-4600). In the matched tumour/germline samples, somatic acquired mutation identification was performed using a Bayesian algorithm implemented in the tool Seurat^22^. Functional annotation of identified somatic mutations was performed with the tool SnpEff 23. Copy Number Variation (CNV) in matched pairs WGS files was determined with the tool Control-FREEC^24^.

### RNAseq

Cell lines were kept under standard cell culture conditions FLO1 and MFD1 were cultured in DMEM supplemented with 10% FBS and antibiotics. OE33 were cultured in RPMI complete media 10% and antibiotics. Cells seeded in 10 cm^2^ dishes, three replicates for each cell line were grown for 72 hours. RNA extraction was performed following manufacturer recommendations (Reliaprep, Promega). RNA QC and libraries were processed by Cambridge Genomics Service in a NextSeq500 Illumina Platform. Approximately 20 million reads library per replicate were mapped using Tophat against hg19^25^. Reads were transformed to absolute counts using default settings in HTseq^26^, relative expression between samples and within replicates are displayed as logarithm_2_Counts per Million (logCPM) following the algorithms implemented in EdgeR^27^. For the RNAseq analysis reads were first trimmed to remove Illumina adapter sequence using trimmomatic 0.32 (Bolger *et al.*^28^). Trimmed reads were aligned to the ensemble transcription (release 72) human genome 19 (hg19) using the RNA-Star aligner (version 2.3.0e) (Dobin *et al.*, 2013). Differential expression analysis was carried out using Cuffdiff (Trapnell *et al.*^25^) in default settings.

### Assessment of Clonality in MFD-1

Cells were seeded at a density of 1000 cells in 10 ml of DMEM complete media in 10 cm^2^ petri dishes and maintained under standard cell culture conditions until colonies were observed. Colonies were picked using a ring cloning method^29^ and transferred to a new container for further expansion. Mutations identified in the parent cell line were screened in three colonies using Sanger sequencing.

### Organotypic culture

3D oesophageal organotypic cultures were performed as previously described^15^,^30^,^31^. The 3D cultures were kept under incubation for 14 days, then harvested, fixed in 4% paraformaldehyde and submitted for histopathological processing and Haematoxylin and Eosin staining.

### Tumour xenografts

Female SCID/NOD mice were inoculated with epithelial cells (4 million) alone or epithelial cells (4 million) embedded with normal or cancer associated fibroblasts (2 million). Inoculation of cells proceeded as follows: In the left flank epithelial cells only, in the middle epithelial cells mixed with normal oesophageal fibroblasts and in the right flank epithelial cells mixed with cancer associated fibroblast from the oesophagus^15^. Cells were inoculated in a volume of 150 **μ**l PBS. OE33 and FLO-1 cells were used as controls. Mice were culled at day 64 post inoculation, tumours were measured and fixed in paraformaldehyde for histology documentation.

### Transposase-accessible chromatin with highthroughput-sequencing (ATAC-seq)

ATAC-seq reports on the accessible regions of the genome which are thought to represent areas of “active” chromatin^32^,^33^. Of the nuclei from fifty thousand cells from MFD-1, OE33 and the non-neoplastic oesophageal cell line HET1A was the input material in an ATAC-seq protocol^32^,^33^ which involves treatment of a nucleic fraction with hyperactive Tn5 transposase that simultaneously cut and ligate adapters. This was followed by 12 cycles of PCR amplification and purification of amplified products was made with 1.8 volumes of SPRI beads (Ampure XL) following manufacturer recommendations. Library QC was performed with the qubit assay. One microliter of the library was used on the TapeStation D1000 screentapes. Library stocks were normalized to 0.5 pg/**μ**l and analysed by qPCR using the Illumina quantification kit (KAPA Biosystems) in triplicate. The library concentration was calculated using the equation in the KAPA kit instructions which uses the size of the library as calculated by the Tapestation, and multiplied by the dilution factor used to make a final 10 nM stock. ATAC-seq libraries (12 pM) were loaded onto a HiSeq-2500 with a 1% PhiX spike on a Paired End 101 × 101, dual indexed run.

### ATAC-seq data analysis

Raw fastq files were de-multiplexed and trimmed using Trimmomatic (version 0.32)^28^. Trimmed reads were aligned to hg19 using Bowtie2^34^ with flags –X2000 –dovetail. Mapped reads were filtered to remove unpaired and low mapping quality reads (<q30) using samtools^35^. Optical duplicates were removed with Picard and finally reads mapping to encode blacklist regions were removed^36^. Next, peak calling was carried out using MACS2^37^ using flags -q 0.01 -g hs -f BAM –nomodel –shift -75 –extsize 150 -B –SPMR. The shift and extend is to focus the peak calling on Tn5 cleavage sites (5′ end of the read). Differentially accessible regions were identified by merging bam files of the conditions to be compared and recalling peaks using MACS2^37^. A 500 bp window around the summit of the top 50,000 regions identified by MACS2 were analysed for differential accessibility using Cufflinks^25^. Normalised cleavage events across the differentially accessible regions were then counted using HOMER^38^. Heatmaps were ploted with the tool GENE-E (BROAD Institute) De novo motif discovery was carried out in HOMER^38^ with flag –cpg for background normalisation. Data are deposited with ArrayExpress (Accession number E-MTAB-4209).

## Results

### Genetic confirmation of MFD-1 derivation from parent tumour tissue

**Figure 1:**
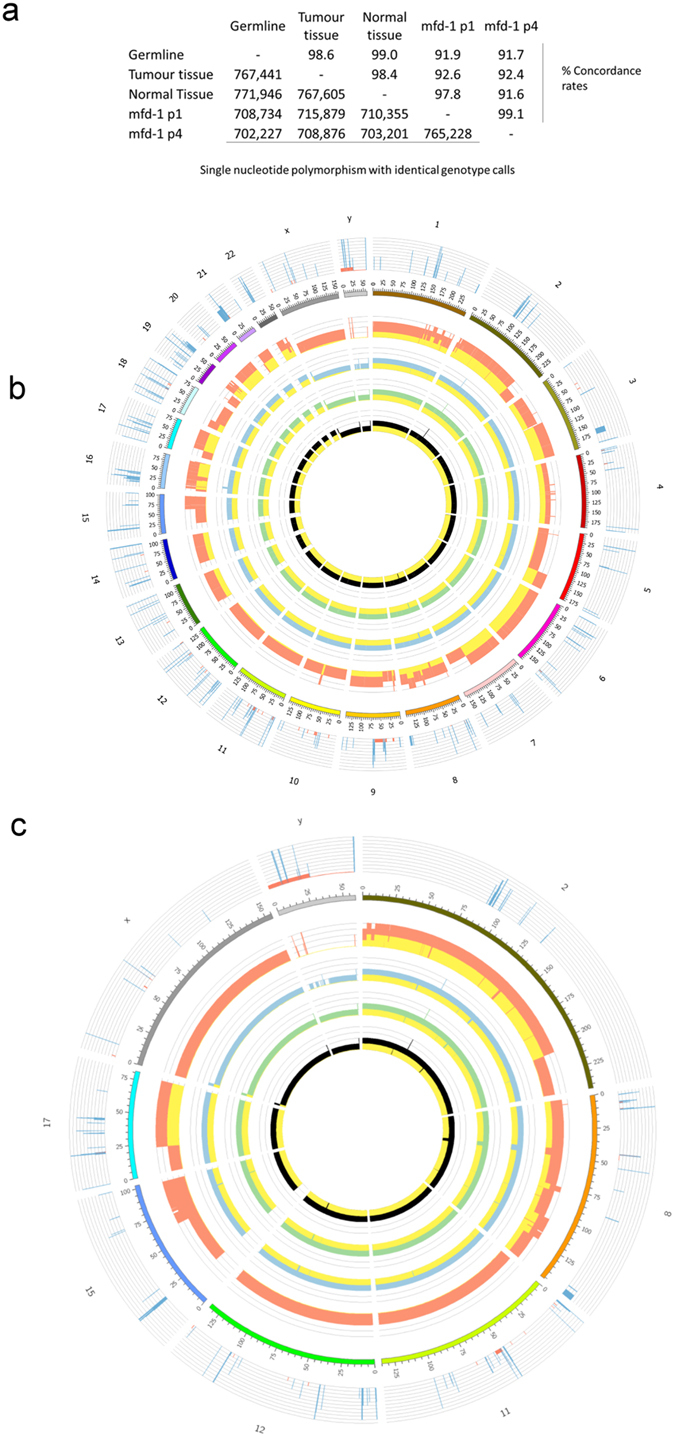
Whole genome SNP genotyping and landscape of copy number variations in normal, tumour and MFD-1 genome. (**a**) Genetic material extracted from germ line, tumour, adjacent normal tissue, and two passages of the MFD-1 cell lines were analysed with the SNP6 array platform. The maximum number of SNPs with identical genotype calls was observed between genomic material isolated from normal tissue and that extracted from peripheral blood cells reaching 771,946 for a 99% concordance rate (Genomic DNA from PBMCs), (**b**,**c**) Circos plots with Genomic CNVs on the inner 4 rings (Black: genomic material from PBMCs, Green: Genomic material from normal oesophagus, Blue: genomic material from tumour, Orange: genomic material from MFD-1) and WGS data from the parent tumour on the outer ring. (**b**) Whole genome (**c**) chromosomes 2, 8, 11, 12, 15, 17, X and Y. CNV comparison between germ-line, tumour and cell line DNA confirms derivation of MFD-1 from the parent tumour. For example, the CNV plot of MFD-1 shows a loss of the Y chromosome and a duplication of the X chromosome that was also revealed by WGS in tumour DNA. The MFD-1 cell line retains CNV from germline DNA and tumour DNA. CNVs found in the normal genome, such as the gain with a scale of 4 on chromosome 2 at 95 cM is readily observed in all of the genomes under analysis, acting as a fingerprint of identity. A homozygous deletion is observed in genome isolated from PMBC on chromosome 8 at 45 cM, this mark is preserved across the genomes under analysis. On chromosome 16 at 20 cM there is a gain CNV in the tumour tissue that is not found in the normal genome (PMBC or adjacent normal tissue DNA) but is readily observed in the MFD-1 cell line.

Analysis of genotypic and Copy Number Variation (CNV) data from the SNP6 platform confirmed the shared, parental origins of DNA from PBMCs, normal oesophagus, oesophageal cancer and two passages (P1 & P4) of the MFD-1 cell line (Fig. 1a–c). To allow visualisation of the size and location of genomic CNVs across samples data from the SNP6 platform were combined with WGS from the parent tumour and represented diagrammatically using Circos plots^39^ (Fig. 1b,c). Analysis of the SNP6 data with the PICNIC algorithm showed that the MFD-1 cell line is likely to harbour a triploid chromosome complement (Supplementary Figure 1).

### Identification of somatic acquired mutations in parent tumour tissue using WGS

**Figure 2:**
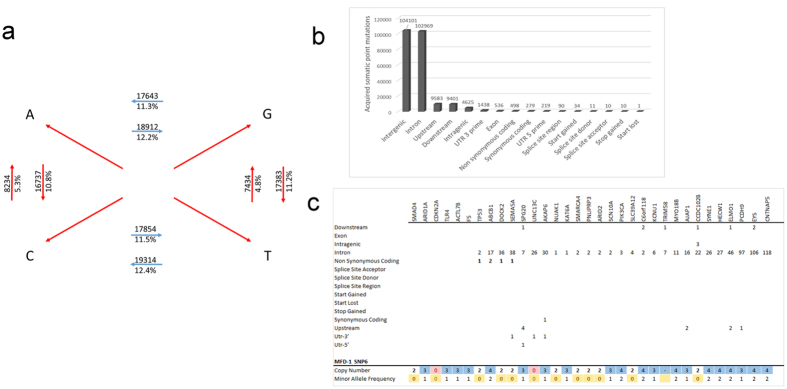
Somatic acquired mutations in tumour and copy number in MFD-1 . (**a**) Transition and transversion frequency of somatic acquired mutations in tumour tissue. (**b**) Histogram of somatic acquired mutations in tumour. A left skew histogram shows the most common mutations are in non-coding region and the less common but functionally relevant mutations are in regions around or within genes. (**c**) Recurrent and frequent genes mutated in oesophageal adenocarcinoma were assessed in the dataset of acquired mutations catalogued in the tumour. Genomic regions and frequency of somatic acquired mutations is shown for each gene. In this panel four genes have non-synonymous acquired mutations. The ABCB1 carries two whilst TP53, DOCK2 and SEMA5A carry one. The lower frame shows MFD-1 copy number status revealed by SNP-6 array platform and the minor allele frequency. The colour code represents the aneuploidy status with yellow indicating loss of heterozygosity, red homozygous deletion and blue copy number gain. The ABCB1 has a copy number state of 4 compatible with amplification whilst the TP53, SEMA5A and DOCK2 shows loss of heterozygosity with a minor allele frequency of zero and duplication of a mutant allele which translate as a copy number of 2. This is suggestive of loss of heterozygosity in a copy neutral state.

Using a Bayesian algorithm in matched paired genomes^22^ a somatic single nucleotide variant (SNV) was observed every 19,932 bases in the parent tumour, in keeping with the high mutational burden of OAC^4^. The number of transitions and transversions were evenly distributed in this tumour with 73,723 and 81,860 respectively for a ratio TS/TV of 0.906 (Fig. 2a) and most SNVs were found in non-coding regions (Fig. 2b). For the purposes of MFD-1 validation, genes with frequent and recurrent mutations in oesophageal adenocarcinoma, as determined by the most recent WGS studies^3^,^4^, were assessed in the dataset of single nucleotide acquired mutations identified in the parent tumour. Of 33 candidate genes, four were found to have non-synonymous acquired mutations with a potential high impact on product function in the parent tumour. These genes were ABCB1, DOCK2, SEMA5A and TP53 (Fig. 2c) and ABCB1 contained 2 separate non-synonymous coding mutations. Furthermore these genes are in regions with major structural abnormalities revealed by the CNV annotation. For example, the ABCB1 gene is in a region with a copy number state of four, whilst DOCK2, SEMA5A and TP53 loci are in regions subjected to loss of heterozygosity in a copy neutral state which translates to a duplication of the mutant allele (Fig. 2c).

### Genotyping of acquired mutations in MFD-1

**Figure 3:**
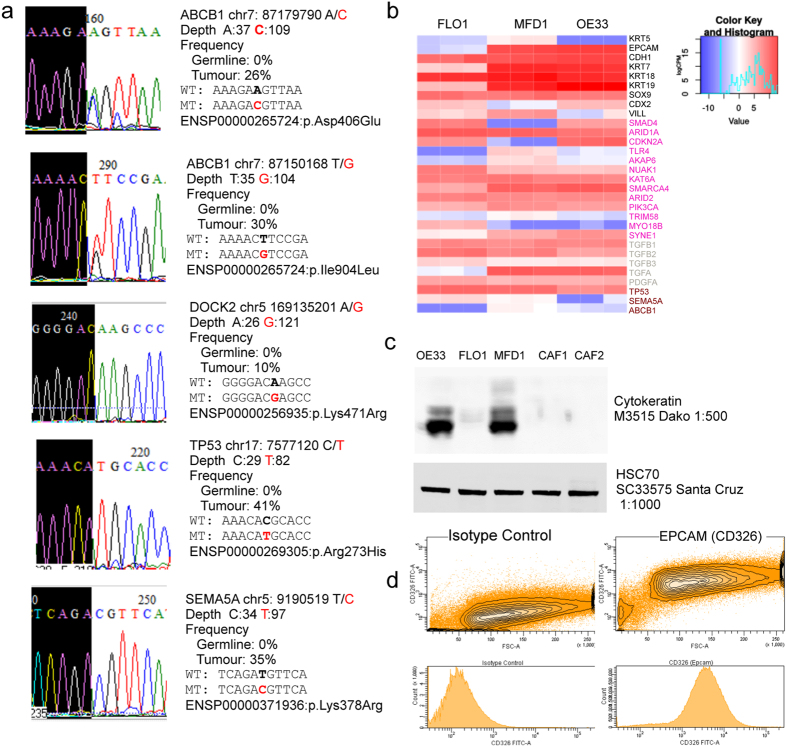
Selected validation of the mutation and expression profile of MFD-1 using multiple platforms. (**a**) Sequencing of genomic DNA from the MFD-1 cell line. PCR primers designed ~250 bp around the mutation were created at the following positions: ABCB1 (Chr7: 87179790 and 87150168); DOCK2: Chr5: 169135201; TP53: Chr17: 7577120; SEMA5A: Chr5: 9190519. The mutant allele is highlighted in red. The sequence depth in the germline and tumour tissue, the frequency observed for the mutant allele in NGS reads, the sequence adjacent to the observed mutation and the Ensemble identification reference is presented. The MFD1 cell line is heterozygote at two somatic acquired mutations on the ABCB1 gene, homozygote wild-type in the DOCK2 gene, and homozygote mutant in the TP53 and SEMA5A gene. (**b**) Expression profiles shown as logCPM in epithelial markers and common recurrent genes in OAC. Dark blue counts not found in RNAseq dataset. (**c**) Pan cytokeratin western blot in lysates from OE33, FLO-1, MFD-1 and two Cancer associated fibroblast. (**d**) EPCAM (CD326) stained using flow cytometry in MFD-1.

To confirm the retention of these potentially important mutations in MFD-1 genomic DNA from the MFD-1 cell line and three derived clones obtained by single cell dilution ring cloning method^29^ was genotyped by Sanger sequencing at the 5 acquired mutations identified by WGS in the tumour. Four of the five somatic acquired mutations identified by WGS in the parent tumour tissue were found in the MFD-1 cell line and the genotype calls were identical in the parent MFD-1 cell line and derived clones (Fig. 3a). The DOCK2 somatic acquired mutation on chromosome 5 at genomic position 169135201, the mutant allele G, runs at a frequency of 10% of the reads by WGS but the cell line is homozygous wild type for the reference allele A. Genotyping of the ABCB1 somatic acquired mutation in the cell line or derived clones is consistent with a heterozygous state. This gene on chromosome 7 carries two somatic acquired mutations at positions 87179790 and 87150168 with a frequency in the tumour of 26% and 30% of the WGS reads respectively (Fig. 3a). TP53 on chromosome 17 carries a mutation at position 7577120 found in 41% of the WGS reads in the tumour, the MFD-1 parent line and derived clones are homozygote mutant. Finally the SEMA5A gene on chromosome 5 at position 9190519 carries a mutation in the tumour found in 35% of the WGS reads whilst the MFD-1 parent cell line and clones are homozygotes mutant (Fig. 3a). By comparison two of the most commonly used OAC cell lines in current research, FLO1 and OE33, do not carry these mutations (Data not shown).

### Comparative analysis of gene expression between OAC cell lines using RNA-seq

Previous reports have documented the use of various platforms to confirm expression of gene products related to the expected morphology of OAC cells. For instance, verification of the OANC1 cell line was performed using immunohistochemistry for columnar (CK18), glandular (CK7) and intestinal (CDX2, SOX9, villin) markers in xenografts. In keeping with the genome wide approach used to document somatic mutations in the MFD-1 parent tumour, we performed RNA-seq of MFD-1, OE33 and FLO1 in culture to compare global gene expression (Fig. 3b) (the authenticity of OE33 and FLO1 was first confirmed by short tandem repeat analysis; data not shown). MFD-1 was confirmed to express CK18 (KRT18), CK7 (KRT7), SOX9 and Villin in high abundance. CDX2 was also expressed but at lower levels when compared to OE33 and FLO1. FLO1 appeared to have relatively little expression of mRNA coding for cytokeratins, except CK18 (KRT18) compared to OE33 and MFD-1 (Fig. 3b). This relative lack of expression was confirmed at the protein level using a pan-CK antibody in Western blot (Fig. 3c). Interestingly FLO1 showed very low expression of EpCAM mRNA in contrast to OE33 and MFD-1 and the majority of primary oesophageal tumours. EpCAM expression at the cell membrane was confirmed in MFD-1 by flow cytometry (Fig. 3d). Integrity of expression of components the TGF family, used previously to authenticate another OAC line, SK-GT-4, was confirmed in all 3 cell lines (Fig. 3b, grey labels)

Sixteen of the 33 recurrently mutated genes in OAC had detectable mRNA expression in the 3 cell lines studied (Fig. 3b). In the MFD-1 cell line there was no expression of CDKN2A, consistent with a homozygous deletion at chromosome 9 in the SNP6 array data (Figs 3b and 1b). MFD-1 was the only cell line of those tested to express both SEMA5A and ABCB1 (Fig. 3b).

### ATAC-seq analysis of the open accessible chromatin landscape of MFD-1 cells

**Figure 4:**
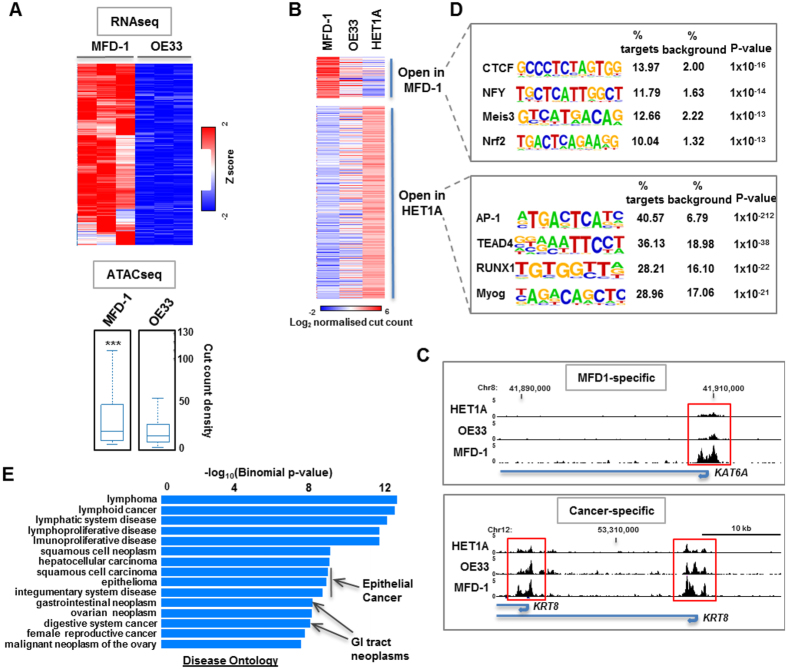
Open chromatin regions in MFD-1 cells. (**a**) RNA-seq analysis (top) of genes differentially expressed to higher levels in MFD-1 compared to OE33 cells (>3 fold change; P-value < 0.01). Each column represents one biological replicate. Data are row Z-normalised. The corresponding ATAC-seq signal in a 700 bp window around the TSS (−500 to +200 bp) of this cohort of genes in each cell line is shown as a boxplot of cut count densities (bottom). ***P-value < 0.05 (2 × 10^−13^). (**b**) ATAC-seq analysis showing the cut counts in regions showing differential accessibility (>3 fold; P-value < 0.05) between MFD-1 and HET1A cells. Data are shown for MFD-1, OE33 and HET1A cells and grouped according to being more open in MFD-1 or HET1A cells. (**c**) UCSC genome browser tracks showing ATAC-seq cleavage data associated with the KAT6A (top) and KRT8 (bottom) loci in HET1A, OE33 and MFD-1 cells. Regions of open chromatin associated with the TSS (arrows) are boxed. (**d**) De novo motif discovery of transcription factor binding sites over-represented in the regions that are either open in MFD-1 cells (top) of MET1A cells (bottom). (**e**) GO term analysis of genes associated with a TSS showing changes (>3 fold) in open chromatin in MFD-1 compared to HET1A cells. The most highly significant terms associated with disease Ontology are shown.

A second approach to characterise the MFD-1 cell line was the assay for transposase-accessible chromatin using sequencing (ATAC-seq) analysis^32^,^33^. This technique reports on the accessible regions of the genome, which are thought to represent areas of “active” chromatin. MFD-1 was compared directly with OE33 and not FLO1, as FLO1 had been shown to express few oesophageal related epithelial markers (Fig. 3b,c). First we identified all of the genes that were expressed to significantly higher levels in MFD-1 compared to OE33 cells (Fig. 4a, top). Next we performed ATAC-seq on MFD-1 and OE33 cells and extracted the chromatin accessibility data (transposase cut counts) around the transcription start sites (TSS) of the genes showing elevated mRNA expression in MFD-1 compared to OE33 cells. The distribution of cut count densities showed that the chromatin accessibility around these genes was significantly higher in MFD1 cells (Fig. 4a, bottom), in keeping with the notion that ATAC-seq reports on “active chromatin”. Next, we investigated which regions are more open in cancer cells and whether these differed between MFD1 and OE33 OAC cell lines. We compared the ATAC-seq signals in MFD-1 and OE33 OAC cells to HET1A cells, which were derived from non-cancerous oesophageal tissue. We identified two classes of regions, which showed >3 fold changes in chromatin accessibility which were either higher (open in MFD-1) or lower (open in HET1A) in MFD-1 compared to HET1A cells (Fig. 4b). While MFD1 cells showed clear differences to the HET1A cells, OE33 cells showed an intermediate pattern, demonstrating the heterogeneity between these OAC-derived cell types. For example, the *KAT6A* promoter is more open specifically in MFD-1 cells (Fig. 4c, top) although at other loci, exemplified by the *KRT8* gene, the chromatin associated with the TSS is more open in both MFD-1 and OE33 cancer cell lines compared to HET1A cells (Fig. 4c, bottom). These results are in keeping with the different gene expression profiles exhibited by these cells (Fig. 3b). To gain potential insights into the regulatory processes that are altered in MFD-1 cells, we searched for over-represented transcription factor binding motifs within the regions of chromatin exhibiting differential accessibility. In regions of chromatin activated in MFD1 cells, motifs recognised by CTCF, NFY, Meis3 and Nrf2 were identified (Fig. 4d, top). In contrast, in chromatin regions showing reduced accessibility in MFD-1 cells and hence potentially lower activity, a different set of motifs were identified with AP-1 figuring most prominently among these (Fig. 4d, bottom). Thus regulatory events controlled by different transcription factors are likely important determinants of the gene expression programmes in MFD-1 and HET1A cells. Interestingly, Gene Ontology analysis of the genes associated with the regulatory regions exhibiting differential accessibility (either increased or decreased) in MFD-1 cells showed enrichments for a large number of terms associated with cancer, including several epithelial cancers and GI tract neoplasms (Fig. 4e).

### *In vitro* and *in vivo* analysis of MFD-1 as a model OAC system

**Figure 5:**
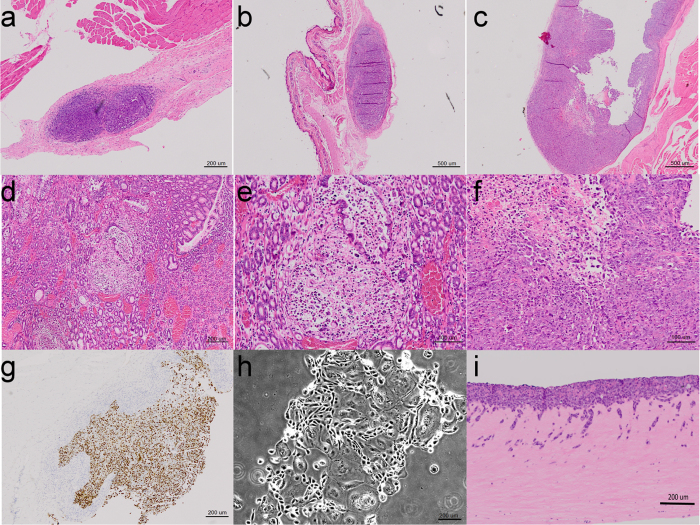
MFD-1 is tumour forming in SCID mouse. (**a**) MFD-1 cell line tumour in SCID mouse 4X (**b**) Tumour in SCID mouse from MFD-1 cells implanted with Normal oesophageal fibroblast 2X. (**c**) Tumour in SCID mouse from MFD-1 cells implanted with Cancer Associated Fibroblast (CAFs) 2X (**d**) Tumour H&E from case 4X (e) Tumour H&E from case 10X (**f**) Tumour in SCID mouse from MFD-1 cells implanted with Cancer Associated Fibroblast 10X (**g**) KI-67 of tumour in SCID mouse from MFD-1 cells implanted with CAFs 4X (**h**) MFD-1 cell line morphology under live microscopy 4X (**i**) MFD-1 in 3D cultures.

To enable the rational design and testing of new therapeutic options for OAC new model systems such as MFD-1 need not only to represent the parent tumour at a genetic level, but furthermore recapitulate tumour growth *in vivo* and respond to microenvironmental signals that are increasingly recognised as determinants of cancer outcome^15^. MFD-1 was implanted in SCID mice alone (Fig. 5a) or in combination with normal oesophageal fibroblasts (Fig. 5b) or cancer associated fibroblasts (CAF) (Fig. 5c). In keeping with our previous findings in OAC^15^, when MFD-1 cells were combined with CAF xenograft tumours grew more quickly and to a larger size. When compared by routine H&E histology, the CAF containing tumours shared a similar morphology to the parent tumour (Fig. 5d–f) and were proliferative (Fig. 5g). Of note, the established OAC cell lines FLO1 and OE33 were implanted in an identical way in SCID mice in the same experiment; OE33 was non-tumourigenic under all conditions and FLO1 only formed tumours when combined with CAF, suggesting that MFD-1 is a more representative line for the invasive OAC phenotype.

We have previously demonstrated that selected OAC cell lines are invasive in a range of *in vitro* assays in response to microenvironmental stimuli^15^. We therefore tested MFD-1 using these assays. MFD-1 has been maintained under routine culture conditions for >35 passages and retains characteristic morphological features (Fig. 5h). MFD-1 was invasive in 3D organotypic culture (Fig. 5i) and in Transwell invasion assays (data not shown). The somatic acquired mutations identified in TP53 and SEMA5A in MFD-1 (Fig. 3a) were present in the explanted xenografts confirming the cell line of origin (data not shown).

## Discussion

Research in OAC is hampered by a lack of pre-clinical models. This deficiency is frustrating efforts to understand the basic biology of progression to OAC and the development of novel therapies for established cancer^7^. Improved understanding of the human genome^40^ has suggested a need for therapeutic agents targeting specific driver mutations. However, standard cell-lines match patient tumours poorly at a molecular level^12^ and do not fully recapitulate clinical drug responsiveness^41^; this has led to derivation of cells *in vitro* as primary cell-lines^42^,^43^ or organoids^44^, or as patient-derived xenografts (PDXs)^43^,^45^,^46^,^47^,^48^. Such cells are urgently required for testing targeted agents for treatment of OAC which is currently dependent on a small set of historic cell-lines. Here, we have described the first fully-characterised system (MFD-1) to arise from the oesophageal International Cancer Genome Consortium project and confirmed that it represents an invasive tumour model containing somatic mutations in genes known to be important in OAC biology that correspond to mutations in the parent tumour. We also offer further characterisation of two commonly used historical lines, OE33 and FLO1.

The majority of cell lines currently used in OAC research are decades old and were not subjected to the kind of detailed genetic authentication that current technologies make possible. For example OE33 was derived from a stage IIa Barrett’s cancer in 1996 and was subjected to karyotyping and cell surface antigen phenotyping, but no analysis of the primary tumour was presented, so the research community cannot know if the mutational burden of OE33 is representative of the tumour of origin^49^. Subsequent short tandem repeat (STR) comparison of OE33 with the tumour of origin taken from formalin fixed paraffin embedded tissue has confirmed the authenticity of current OE33 cell stocks, but no genome wide analysis of mutational status has been performed^11^. Similar problems exist for all historic OAC cell lines. More recently Clemons and colleagues developed the OANC1 cell line^14^. Authenticity was confirmed by STR comparing the cell line and tumour of origin at 16 loci and targeted deep sequencing of 48 cancer related genes was performed, but only in the cell line. OANC1 is a valuable new addition for OAC researchers, however it may represent a “non-classical” OAC as it was derived from a 44-year-old patient and more information about the tumour of origin is required.

By contrast, the strength of the current work is that we have used multiple genome-wide platforms (WGS, SNP arrays, ATAC-seq and RNA-seq) in the primary tumour and the MFD-1 cell line to confirm the tissue of origin and to accurately document somatic mutations that can be further tested as potential tractable targets. Our initial analysis has concentrated on a panel of genes commonly mutated in OAC constructed by synthesizing data from the WGS studies described by Dulak^3^ and Weaver^4^, but this does not preclude future interrogation of the data as new targets are discovered.

We describe 5 mutations in 4 genes (*ABCB1, DOCK2, SEMA5A* and *TP53*) with relevance to OAC found in the parent tumour by WGS. Interestingly, the mutation observed in the lowest number of reads from the WGS data (*DOCK2*, mutant allele 10%) is homozygous wild-type in MFD-1, suggesting that the cell line is likely derived from the dominant clone in the tumour. The finding of a mutation in *TP53* that has been extensively documented in OAC suggests that MFD-1 is representative of primary OAC tumours. The mutations in *ABCB1* and *SEMA5A* described here have not been previously reported in oesophageal cancer. Importantly, the products of these genes are expressed in MFD-1 making the cell line a useful model to study the biological effects of these mutations. *ABCB1* encodes one of a family of ATP-binding cassette membrane transporters that efflux many chemically diverse compounds across the plasma membrane. These transporters are implicated in the development of multidrug resistance in cancer cells treated with chemotherapy (reviewed in ref. 50). *SEMA5A* is a Semaphorin important in neuronal development that has recently been implicated in cancer development by influencing angiogenesis, tumour growth and metastasis (reviewed in ref. 51). The coexistence of mutations in *SEMA5A* and *ABCB1* and the expression of both genes together in MFD-1 is of particular relevance because mutations in *SEMA5A* and *ABCB1* were the only mutations found to co-occur more often than would be expected by chance in a cohort of 112 cases (P = 0.0021) of OAC by Weaver et.al, but the reasons for this association remain unclear^4^. In addition, the *ABCB1* mutations may indicate a potential mechanism for chemotherapy resistance that was observed in the primary tumour. None of these mutations were found in OE33 or FLO1 and neither of these lines express ABCB1 and SEMA5A together. MFD-1 was generated from a tumour in a patient who had been treated with neoadjuvant chemotherapy. This leads to the potential that some of the mutations in MFD-1 may be chemotherapy induced, however the frequency of mutations observed in *SEMA5A* and *ABCB1* in previous studies of chemotherapy naïve OAC point to these mutations being tumour specific.

MFD-1 is tumourigenic in SCID mice. This adds substantially to the utility of MFD-1 as a pre-clinical model of OAC and enables complex experiments that may include other constituents of the tumour microenvironment and/or manipulation of the host organism to mimic human treatments, such as novel CAF targeted agents or combination therapies. Similarly to the results reported by Clemons *et al.*^14^ we found that FLO1 and OE33 are poorly tumourigenic in SCID mice, this is somewhat surprising given that OE33 is one of the most commonly used OAC cell lines in xenograft experiments, however these findings reinforce the tumourigenic nature of MFD-1.

MFD-1 is derived from a single clone from the parent tumour and has to date appeared stable *in vitro*. This is advantageous as a molecular tool for genetic manipulation of candidate genes, but has the disadvantage of not offering insight into the heterogeneity of OAC. More complex models that recapitulate the complexity of OAC *in vivo*, such as primary xenografts or spheroids, will be required to address this issue. In addition, MFD-1 was derived from a particularly aggressive tumour and is the only cell line to be developed in our laboratory from several attempts (>10). This highlights the difficulty in performing research in OAC with the currently available pre-clinical models that may not be representative of tumours seen in the clinic. However, MFD-1 does contain mutations that have been observed in commonly mutated genes in OAC from large, contemporary cohorts^3^,^4^,^21^ and this is reassuring that MFD-1 has relevance for future research.

In summary, we describe a pre-clinical model system developed from the oesophageal ICGC project. The availability of genome and transcriptome wide data from the parent tumour and cell line makes MFD-1 perhaps the best-catalogued OAC cell line in existence. Future OAC researchers will have confidence in the integrity of work performed with this cell line and will be able to easily confirm the significance of genetic changes in MFD-1 by reference to the parent tumour by examination of the WGS data. Research in OAC is held back due to a lack of hi-fidelity pre-clinical models and further efforts should be directed to bridge this technology gap.

## Additional Information

**How to cite this article**: Garcia, E. *et al.* Authentication and characterisation of a new oesophageal adenocarcinoma cell line: MFD-1. *Sci. Rep.* **6**, 32417; doi: 10.1038/srep32417 (2016).

## Supplementary Material

Supplementary Information

## References

[b1] Noble F. *et al.* Refining pathological evaluation of neoadjuvant therapy for adenocarcinoma of the esophagus. World J. Gastroenterol. 19, 9282–9293 (2013). 24409055 10.3748/wjg.v19.i48.9282 PMC3882401

[b2] Vaughan T. L. & Fitzgerald R. C. Precision prevention of oesophageal adenocarcinoma. Nature reviews. Gastroenterology & hepatology 12, 243–248, doi: 10.1038/nrgastro.2015.24 (2015). 25666644 PMC4382373

[b3] Dulak A. M. *et al.* Exome and whole-genome sequencing of esophageal adenocarcinoma identifies recurrent driver events and mutational complexity. Nat. Genet. 45, 478–486 (2013). 23525077 10.1038/ng.2591 PMC3678719

[b4] Weaver J. M. *et al.* Ordering of mutations in preinvasive disease stages of esophageal carcinogenesis. Nat. Genet. 46, 837–843 (2014). 24952744 10.1038/ng.3013 PMC4116294

[b5] Nones K. *et al.* Genomic catastrophes frequently arise in esophageal adenocarcinoma and drive tumorigenesis. Nat Commun 5, 5224, doi: 10.1038/ncomms6224 (2014). 25351503 PMC4596003

[b6] Murugaesu N. *et al.* Tracking the genomic evolution of esophageal adenocarcinoma through neoadjuvant chemotherapy. Cancer discovery 5, 821–831, doi: 10.1158/2159-8290.cd-15-0412 (2015). 26003801 PMC4529488

[b7] Boonstra J. J., Tilanus H. W. & Dinjens W. N. Translational research on esophageal adenocarcinoma: from cell line to clinic. Dis.Esophagus. 28, 90–96 (2015). 23795680 10.1111/dote.12095

[b8] Rubio C. A. *et al.* Mucous gland metaplasia in the esophagus and gastric mucosa in baboons. Anticancer Res. 31, 2187–2190 (2011). 21737639 PMC3468912

[b9] Attwood S. E., Harrison L. A., Preston S. L. & Jankowski J. A. Esophageal adenocarcinoma in “mice and men”: back to basics! Am. J. Gastroenterol. 103, 2367–2372 (2008). 18844624 10.1111/j.1572-0241.2008.02004.x

[b10] Garman K. S., Orlando R. C. & Chen X. Review: Experimental models for Barrett’s esophagus and esophageal adenocarcinoma. Am. J. Physiol Gastrointest. Liver Physiol 302, G1231–G1243 (2012). 22421618 10.1152/ajpgi.00509.2011 PMC4380479

[b11] Boonstra J. J. *et al.* Verification and unmasking of widely used human esophageal adenocarcinoma cell lines. J. Natl. Cancer Inst. 102, 271–274 (2010). 20075370 10.1093/jnci/djp499 PMC2902814

[b12] Gillet J.-P. *et al.* Redefining the relevance of established cancer cell lines to the study of mechanisms of clinical anti-cancer drug resistance. Proceedings of the National Academy of Sciences of the United States of America 108, 18708–18713, doi: 10.1073/pnas.1111840108 (2011). 22068913 PMC3219108

[b13] Zhang X. *et al.* Malignant transformation of non-neoplastic Barrett’s epithelial cells through well-defined genetic manipulations. PLoS. One. 5 (2010). 10.1371/journal.pone.0013093 PMC2948040 20927195

[b14] Clemons N. J. *et al.* Characterization of a novel tumorigenic esophageal adenocarcinoma cell line: OANC1. Dig. Dis. Sci. 59, 78–88 (2014). 24077944 10.1007/s10620-013-2882-8

[b15] Underwood T. J. *et al.* Cancer Associated Fibroblasts Predict for Poor Outcome and Promote Periostin-Dependent Invasion in Oesophageal Adenocarcinoma. The Journal of pathology, doi: 10.1002/path.4467 (2014). PMC4312957 25345775

[b16] Leedham S. J. *et al.* Individual crypt genetic heterogeneity and the origin of metaplastic glandular epithelium in human Barrett’s oesophagus. Gut 57, 1041–1048 (2008). 18305067 10.1136/gut.2007.143339 PMC2564832

[b17] Edge S. B. & Compton C. C. The American Joint Committee on Cancer: the 7th Edition of the AJCC Cancer Staging Manual and the Future of TNM. Annals of Surgical Oncology 17, 1471–1474, doi: 10.1245/s10434-010-0985-4 (2010). 20180029

[b18] Mandard A. M. *et al.* Pathologic assessment of tumor regression after preoperative chemoradiotherapy of esophageal carcinoma. Clinicopathologic correlations. Cancer 73, 2680–2686 (1994). 8194005 10.1002/1097-0142(19940601)73:11<2680::aid-cncr2820731105>3.0.co;2-c

[b19] Korn J. M. *et al.* Integrated genotype calling and association analysis of SNPs, common copy number polymorphisms and rare CNVs. Nat. Genet. 40, 1253–1260 (2008). 18776909 10.1038/ng.237 PMC2756534

[b20] Greenman C. D. *et al.* PICNIC: an algorithm to predict absolute allelic copy number variation with microarray cancer data. Biostatistics. 11, 164–175 (2010). 19837654 10.1093/biostatistics/kxp045 PMC2800165

[b21] Weaver J. M., Ross-Innes C. S. & Fitzgerald R. C. The ‘-omics’ revolution and oesophageal adenocarcinoma. Nature reviews. Gastroenterology & hepatology 11, 19–27, doi: 10.1038/nrgastro.2013.150 (2014). 23982683

[b22] Christoforides A. *et al.* Identification of somatic mutations in cancer through Bayesian-based analysis of sequenced genome pairs. BMC.Genomics 14, 302 (2013). 23642077 10.1186/1471-2164-14-302 PMC3751438

[b23] Cingolani P. *et al.* A program for annotating and predicting the effects of single nucleotide polymorphisms, SnpEff: SNPs in the genome of Drosophila melanogaster strain w1118; iso-2; iso-3. Fly. (Austin.) 6, 80–92 (2012). 22728672 10.4161/fly.19695 PMC3679285

[b24] Boeva V. *et al.* Control-FREEC: a tool for assessing copy number and allelic content using next-generation sequencing data. Bioinformatics 28, 423–425, doi: 10.1093/bioinformatics/btr670 (2012). 22155870 PMC3268243

[b25] Trapnell C. *et al.* Differential gene and transcript expression analysis of RNA-seq experiments with TopHat and Cufflinks. Nature protocols 7, 562–578, doi: 10.1038/nprot.2012.016 (2012). 22383036 PMC3334321

[b26] Anders S., Pyl P. T. & Huber W. HTSeq–a Python framework to work with high-throughput sequencing data. Bioinformatics (Oxford, England) 31, 166–169, doi: 10.1093/bioinformatics/btu638 (2015). PMC4287950 25260700

[b27] Robinson M. D., McCarthy D. J. & Smyth G. K. edgeR: a Bioconductor package for differential expression analysis of digital gene expression data. Bioinformatics (Oxford, England) 26, 139–140, doi: 10.1093/bioinformatics/btp616 (2010). PMC2796818 19910308

[b28] Bolger A. M., Lohse M. & Usadel B. Trimmomatic: a flexible trimmer for Illumina sequence data. Bioinformatics 30, 2114–2120, doi: 10.1093/bioinformatics/btu170 (2014). 24695404 PMC4103590

[b29] Reid L. C. Cloning. Methods in enzymology 58, 152–164 (1979). 423757 10.1016/s0076-6879(79)58133-6

[b30] Underwood T. J. *et al.* A comparison of primary oesophageal squamous epithelial cells with HET-1A in organotypic culture. Biol.Cell 102, 635–644 (2010). 20843300 10.1042/BC20100071

[b31] Moutasim K. A., Nystrom M. L. & Thomas G. J. Cell migration and invasion assays. Methods Mol. Biol. 731, 333–343 (2011). 21516419 10.1007/978-1-61779-080-5_27

[b32] Buenrostro J. D., Giresi P. G., Zaba L. C., Chang H. Y. & Greenleaf W. J. Transposition of native chromatin for fast and sensitive epigenomic profiling of open chromatin, DNA-binding proteins and nucleosome position. Nat Methods 10, 1213–1218, doi: 10.1038/nmeth.2688 (2013). 24097267 PMC3959825

[b33] Buenrostro J. D., Wu B., Chang H. Y. & Greenleaf W. J. ATAC-seq: A Method for Assaying Chromatin Accessibility Genome-Wide. Curr Protoc Mol Biol 109, 21.29.21-29, doi: 10.1002/0471142727.mb2129s109 (2015). PMC4374986 25559105

[b34] Langmead B. & Salzberg S. L. Fast gapped-read alignment with Bowtie 2. Nat Methods 9, 357–359, doi: 10.1038/nmeth.1923 (2012). 22388286 PMC3322381

[b35] Li H. *et al.* The Sequence Alignment/Map format and SAMtools. Bioinformatics 25, 2078–2079, doi: 10.1093/bioinformatics/btp352 (2009). 19505943 PMC2723002

[b36] Consortium E. P. An integrated encyclopedia of DNA elements in the human genome. Nature 489, 57–74, doi: 10.1038/nature11247 (2012). 22955616 PMC3439153

[b37] Zhang Y. *et al.* Model-based analysis of ChIP-Seq (MACS). Genome Biol 9, R137, doi: 10.1186/gb-2008-9-9-r137 (2008). 18798982 PMC2592715

[b38] Heinz S. *et al.* Simple combinations of lineage-determining transcription factors prime cis-regulatory elements required for macrophage and B cell identities. Mol Cell 38, 576–589, doi: 10.1016/j.molcel.2010.05.004 (2010). 20513432 PMC2898526

[b39] Krzywinski M. *et al.* Circos: An information aesthetic for comparative genomics. Genome Research 19, 1639–1645, doi: 10.1101/gr.092759.109 (2009). 19541911 PMC2752132

[b40] Chin L., Andersen J. N. & Futreal P. A. Cancer genomics: from discovery science to personalized medicine. Nature medicine 17, 297–303, doi: 10.1038/nm.2323 (2011). 21383744

[b41] Johnson J. I. *et al.* Relationships between drug activity in NCI preclinical *in vitro* and *in vivo* models and early clinical trials. British journal of cancer 84, 1424–1431, doi: 10.1054/bjoc.2001.1796 (2001). 11355958 PMC2363645

[b42] Liu X. *et al.* ROCK inhibitor and feeder cells induce the conditional reprogramming of epithelial cells. The American journal of pathology 180, 599–607, doi: 10.1016/j.ajpath.2011.10.036 (2012). 22189618 PMC3349876

[b43] Onion D. *et al.* Three-Dimensional, Humanised Tumour Growth Assays with Low Passage Tumours Provide a Tumour Microenvironment -Relevant Screen for Novel Chemotherapeutics. NC3Rs workshop: Understanding target-to-function biology in non-clinical oncology research (2014).

[b44] van de Wetering M. *et al.* Prospective derivation of a living organoid biobank of colorectal cancer patients. Cell 161, 933–945, doi: 10.1016/j.cell.2015.03.053 (2015). 25957691 PMC6428276

[b45] Visonneau S., Cesano A., Torosian M. H., Miller E. J. & Santoli D. Growth characteristics and metastatic properties of human breast cancer xenografts in immunodeficient mice. The American journal of pathology 152, 1299–1311 (1998). 9588898 PMC1858587

[b46] Jin K. *et al.* Patient-derived human tumour tissue xenografts in immunodeficient mice: a systematic review. Clinical & translational oncology: official publication of the Federation of Spanish Oncology Societies and of the National Cancer Institute of Mexico 12, 473–480, doi: 10.1007/s12094-010-0540-6 (2010). 20615824

[b47] McKenzie A. *et al.* *In vivo* response and molecular characterisation of a NSCLC squamous cell carcinoma PDX model exhibiting reproducible sensitivity to FGFR inhibitors. Cancer Research 73, 342–342, doi: 10.1158/1538-7445.am2013-342 (2013).

[b48] McKenzie A. *et al.* *In vivo* generation of EGFR-TKI resistance in a patient-derived xenograft (PDX) with an activating EGFR mutation (L858R), and molecular characterisation of resistance mechanisms. Cancer Research 73, doi: 10.1158/1538-7445.am2013-5651 (2013).

[b49] Rockett J. C., Larkin K., Darnton S. J., Morris A. G. & Matthews H. R. Five newly established oesophageal carcinoma cell lines: phenotypic and immunological characterization. British journal of cancer 75, 258–263, doi: 10.1038/bjc.1997.42 (1997). 9010035 PMC2063267

[b50] Shukla S., Ohnuma S. & Ambudkar S. V. Improving cancer chemotherapy with modulators of ABC drug transporters. Curr Drug Targets 12, 621–630 (2011). 21039338 10.2174/138945011795378540 PMC3401946

[b51] Purohit A., Sadanandam A., Myneni P. & Singh R. K. Semaphorin 5A mediated cellular navigation: connecting nervous system and cancer. Biochim Biophys Acta 1846, 485–493, doi: 10.1016/j.bbcan.2014.09.006 (2014). 25263940 PMC4261006

