# Author Correction: Authentication and characterisation of a new oesophageal adenocarcinoma cell line: MFD-1

**DOI:** 10.1038/s41598-018-37591-7

**Published:** 2019-01-22

**Authors:** Edwin Garcia, Annette Hayden, Charles Birts, Edward Britton, Connor Rogerson, Christopher W. Bleaney, Andrew Cowie, Karen Pickard, Massimiliano Mellone, Clarisa Choh, Mathieu Derouet, Patrick Duriez, Fergus Noble, Michael J. White, John N. Primrose, Jonathan C. Strefford, Matthew Rose-Zerilli, Gareth J. Thomas, Yeng Ang, Andrew D. Sharrocks, Rebecca C. Fitzgerald, Timothy J. Underwood, the OCCAMS consortium, Shona MacRae, Nicola Grehan, Zarah Abdullahi, Rachel de la Rue, Ayesha Noorani, Rachael Fels Elliott, Nadeera de Silva, Jan Bornschein, Maria O’Donovan, Gianmarco Contino, Tsun-Po Yang, Hamza Chettouh, Jason Crawte, Barbara Nutzinger, Paul A. W. Edwards, Laura Smith, Ahmad Miremadi, Shalini Malhotra, Alison Cluroe, Richard Hardwick, Jim Davies, Hugo Ford, David Gilligan, Peter Safranek, Andy Hindmarsh, Vijayendran Sujendran, Nick Carroll, Richard Turkington, Stephen J. Hayes, Yeng Ang, Shaun R. Preston, Sarah Oakes, Izhar Bagwan, Vicki Save, Richard J. E. Skipworth, Ted R. Hupp, J. Robert O’Neill, Olga Tucker, Philippe Taniere, Jack Owsley, Charles Crichton, Christian Schusterreiter, Hugh Barr, Neil Shepherd, Oliver Old, Jesper Lagergren, James Gossage, Andrew Davies, Fuju Chang, Janine Zylstra, Grant Sanders, Richard Berrisford, Catherine Harden, David Bunting, Mike Lewis, Ed Cheong, Bhaskar Kumar, Simon L. Parsons, Irshad Soomro, Philip Kaye, John Saunders, Laurence Lovat, Rehan Haidry, Victor Eneh, Laszlo Igali, Ian Welch, Michael Scott, Shamila Sothi, Sari Suortamo, Suzy Lishman, Duncan Beardsmore, Charlotte Anderson, Mike L. Smith, Maria Secrier, Matthew D. Eldridge, Lawrence Bower, Achilleas Achilleos, Andy G. Lynch, Simon Tavare

**Affiliations:** 1 Faculty of Medicine, University of Southampton, Southampton General Hospital, Mailpoint 801, South Academic Block, Tremona Road, Southampton, SO16 6YD United Kingdom; 2 Faculty of Biology, Medicine and Health, Oxford Road, University of Manchester, Manchester, M13 9PT UK; 3 MRC Cancer Unit, University of Cambridge, Hutchison/MRC Research Centre, Box 197, Cambridge Biomedical Campus, Cambridge, CB2 0XZ United Kingdom; 4 Department of Histopathology, Addenbrooke’s Hospital, Cambridge, UK; 5 Oesophago-Gastric Unit, Addenbrooke’s Hospital, Cambridge, UK; 6 Oxford ComLab, University of Oxford, Oxford, UK; 7 Cambridge University Hospitals NHS Foundation Trust, Cambridge, UK; 8 Centre for Cancer Research and Cell Biology, Queen’s University Belfast, Belfast, Northern Ireland UK; 9 Salford Royal NHS Foundation Trust, Salford, UK; 10 Wigan and Leigh NHS Foundation Trust, Wigan, Manchester UK; 11 Royal Surrey County Hospital NHS Foundation Trust, Guildford, UK; 12 Edinburgh Royal Infirmary, Edinburgh, UK; 13 University Hospitals Birmingham NHS Foundation Trust, Birmingham, UK; 14 University Hospital Southampton NHS Foundation Trust, Southampton, UK; 15 Faculty of Medical and Human Sciences, University of Manchester, Manchester, UK; 16 Department of Computer Science, University of Oxford, Oxford, UK; 17 Gloucester Royal Hospital, Gloucester, UK; 18 St Sharrocks’s Hospital, London, UK; 19 Plymouth Hospitals NHS Trust, Plymouth, UK; 20 Norfolk and Norwich University Hospital NHS Foundation Trust, Norwich, UK; 21 Nottingham University Hospitals NHS Trust, Nottingham, UK; 22 University College London, London, UK; 23 Norfolk and Waveney Cellular Pathology Network, Norwich, UK; 24 Wythenshawe Hospital, Manchester, UK; 25 Edinburgh University, Edinburgh, UK; 26 King’s College London, London, UK; 27 Karolinska Institutet, Stockholm, Sweden; 28 University Hospitals Coventry and Warwickshire NHS, Trust, Coventry, UK; 29 Peterborough Hospitals NHS Trust, Peterborough City Hospital, Peterborough, UK; 30 Royal Stoke University Hospital, UHNM NHS Trust, Stoke-on-Trent, UK; 31 Institute of cancer and genomic sciences, University of Birmingham, Birmingham, UK; 32 GI science centre, University of Manchester, Manchester, UK; 33 Cancer Research UK Cambridge Institute, University of Cambridge, Cambridge, UK

Correction to: *Scientific Reports* 10.1038/srep32417, published online 07 September 2016

This Article contains errors.

After publication of the Article it has come to our attention that the cell stocks used for ATAC-seq analyses may have been contaminated. We therefore revalidated the original stocks using STR profiling and repeated ATAC-seq analysis using these new stocks.

Based on their contributions to the repeat experiments, two new authors, Connor Rogerson and Christopher W. Bleaney, should be included in the author list.

As a result, in the Acknowledgements section,

“The authors gratefully acknowledge funding from Cancer Research UK for the OCCAMS/ICGC project, MRC Clinician Scientist Grant for TJU, MCRC CRUK clinical training fellowship for EB, Maria Secrier for her advice regarding the WGS data and Southampton ECMC Tissue Bank and the Southampton Computation modelling group for access to the IRIDIS4 computer resource.”

should read:

“The authors gratefully acknowledge funding from Cancer Research UK for the OCCAMS/ICGC project, MRC Clinician Scientist Grant for TJU, MCRC CRUK clinical training fellowship for EB and CWB, MCRC CRUK non-clinical training fellowship for CR. Maria Secrier for her advice regarding the WGS data and Southampton ECMC Tissue Bank and the Southampton Computation modelling group for access to the IRIDIS4 computer resource.”

Furthermore, in the Author Contributions section,

“Performed the experiments: E.G., A.H., C.B., A.C., K.P., M.M., C.C., M.D., P.D. and M.J.W. Contributed reagent/materials: J.C.S. and R.C.F., OCCAMS Contributed with analysis tools: E.G., E.B., J.C.S., M.R.Z., A.D.S. and R.C.F. OCCAMS prepare figures: E.G., A.H., C.C., E.B. and T.J.U.”

should read:

“Performed the experiments: EG, AH, CB, CWB, AC, KP, MM, CC, MD, PD, MJW. Contributed reagent/materials: JCS, RCF, OCCAMS. Contributed with analysis tools: EG, EB, CR, JCS, MRZ, ADS, RCF, OCCAMS. Prepared figures: EG, AH, CC, EB, CR, TJU.”

The outcome of this analysis differs slightly from the one reported in the Article.

In Materials and Methods section, under the subheading “ATAC-seq data analysis”,

“A 500 bp window around the summit of the top 50,000 regions identified by MACS2 were analysed for differential accessibility using Cufflinks^25^. Normalised cleavage events across the differentially accessible regions were then counted using HOMER^38^. Heatmaps were ploted with the tool GENE-E (BROAD Institute) De novo motif discovery was carried out in HOMER^38^ with flag –cpg for background normalisation. Data are deposited with ArrayExpress (Accession number E-MTAB-4209).”

should read:

“A 500 bp window around the summit of the top 50,000 regions identified by MACS2 were analysed for differential accessibility using Cufflinks^25^. Regions that showed a significant (p < 0.05) linear 5 fold change in ATAC-seq signal were taken forward for further analyses. Normalised cleavage events across the differentially accessible regions were then counted using HOMER^38^. Heatmaps were ploted with the tool GENE-E (BROAD Institute). *De novo* motif discovery was carried out in HOMER^38^ with flag –cpg for background normalisation. Data are deposited with ArrayExpress (Accession numbers E-MTAB-4209 and E-MTAB-6720).”

In Results section, under the subheading “ATAC-seq analysis of the open accessible chromatin landscape of MFD-1 cells”,

“We identified two classes of regions, which showed >3 fold changes in chromatin accessibility which were either higher (open in MFD-1) or lower (open in HET1A) in MFD-1 compared to HET1A cells (Fig. 4b).”

should read:

“We identified two classes of regions, which showed >5 fold changes in chromatin accessibility which were either higher (open in MFD-1) or lower (open in HET1A) in MFD-1 compared to HET1A cells (Figure 4b).”

In the same section,

“For example, the *KAT6A* promoter is more open specifically in MFD-1 cells (Fig. 4c, top) although at other loci, exemplified by the *KRT8* gene, the chromatin associated with the TSS is more open in both MFD-1 and OE33 cancer cell lines compared to HET1A cells (Fig. 4c, bottom).”

should read:

“For example, the *BBOX1* promoter is more open specifically in MFD-1 cells (Figure 4c, top) although at other loci, exemplified by the *KRT8* gene, the chromatin associated with the TSS is more open in both MFD-1 and OE33 cancer cell lines compared to HET1A cells (Figure 4c, bottom).”

and,

“In regions of chromatin activated in MFD1 cells, motifs recognised by CTCF, NFY, Meis3 and Nrf2 were identified (Fig. 4d, top). In contrast, in chromatin regions showing reduced accessibility in MFD-1 cells and hence potentially lower activity, a different set of motifs were identified with AP-1 figuring most prominently among these (Fig. 4d, bottom). Thus regulatory events controlled by different transcription factors are likely important determinants of the gene expression programmes in MFD-1 and HET1A cells. Interestingly, Gene Ontology analysis of the genes associated with the regulatory regions exhibiting differential accessibility (either increased or decreased) in MFD-1 cells showed enrichments for a large number of terms associated with cancer, including several epithelial cancers and GI tract neoplasms (Fig. 4e).”

should read:

“In regions of chromatin activated in MFD1 cells, motifs recognised by FRA1 (AP1), GRHL1, ELF3 and TEAD3 were identified (Figure 4d, top). In contrast, in chromatin regions showing reduced accessibility in MFD-1 cells and hence potentially lower activity, a different set of motifs were identified with JunB (AP-1) figuring most prominently among these (Figure 4d, bottom). Thus regulatory events controlled by different transcription factors are likely important determinants of the gene expression programmes in MFD-1 and HET1A cells. Interestingly, Gene Ontology analysis of the genes associated with the regulatory regions exhibiting increased accessibility in MFD-1 cells showed enrichments for a large number of terms associated with cancer, including several epithelial cancers and GI tract neoplasms (Figure 4e).”

In the legend of Figure 4,

“(**a**) RNA-seq analysis (top) of genes differentially expressed to higher levels in MFD-1 compared to OE33 cells (>3 fold change; P-value < 0.01). Each column represents one biological replicate. Data are row Z-normalised. The corresponding ATAC-seq signal in a 700 bp window around the TSS (−500 to +200 bp) of this cohort of genes in each cell line is shown as a boxplot of cut count densities (bottom). ***P-value < 0.05 (2 × 10^−13^). (**b**) ATAC-seq analysis showing the cut counts in regions showing differential accessibility (>3 fold; P-value < 0.05) between MFD-1 and HET1A cells. Data are shown for MFD-1, OE33 and HET1A cells and grouped according to being more open in MFD-1 or HET1A cells. (**c**) UCSC genome browser tracks showing ATAC-seq cleavage data associated with the KAT6A (top) and KRT8 (bottom) loci in HET1A, OE33 and MFD-1 cells. Regions of open chromatin associated with the TSS (arrows) are boxed. (**d**) De novo motif discovery of transcription factor binding sites over-represented in the regions that are either open in MFD-1 cells (top) of MET1A cells (bottom). (**e**) GO term analysis of genes associated with a TSS showing changes (>3 fold) in open chromatin in MFD-1 compared to HET1A cells. The most highly significant terms associated with disease Ontology are shown.”

should read:

“**(a)** RNA-seq analysis (top) of genes differentially expressed to higher levels in MFD-1 compared to OE33 cells (>3 fold change; P-value < 0.01). Each column represents one biological replicate. Data are row Z-normalised. The corresponding ATAC-seq signal in a 1000 bp window around the TSS (−500 to +500 bp) of this cohort of genes in each cell line is shown as a boxplot of cut count densities (bottom). **** = P-value < 0.0001. **(b)** ATAC-seq analysis showing the cut counts in regions showing differential accessibility (>5 fold; P-value < 0.05) between MFD-1 and HET1A cells. Data are shown for MFD-1, OE33 and HET1A cells and grouped according to being more open in MFD-1 or HET1A cells. **(c)** UCSC genome browser tracks showing ATAC-seq cleavage data associated with the *BBOX1* (top) and *KRT8* (bottom) loci in HET1A, OE33 and MFD-1 cells. Regions of open chromatin associated with the TSS (arrows) are boxed. **(d)** *De novo* motif discovery of transcription factor binding sites over-represented in the regions that are either open in MFD-1 cells (top) or HET1A cells (bottom). **(e)** IPA GO term analysis of genes associated with a TSS showing changes (>5 fold) in open chromatin in MFD-1 compared to HET1A cells. The most highly significant terms associated with disease ontology are shown.”

An updated version of Figure 4 based on the repeated experiments is shown below as Figure 1.

**Figure 1:**
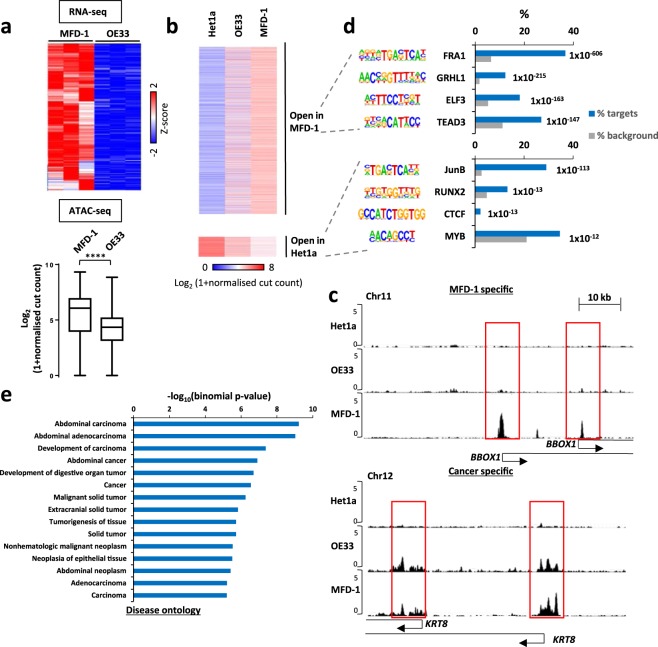
Open chromatin regions in MFD-1 cells. (**a**) RNA-seq analysis (top) of genes differentially expressed to higher levels in MFD-1 compared to OE33 cells (>3 fold change; P-value < 0.01). Each column represents one biological replicate. Data are row Z-normalised. The corresponding ATAC-seq signal in a 1000 bp window around the TSS (−500 to +500 bp) of this cohort of genes in each cell line is shown as a boxplot of cut count densities (bottom). **** = P-value < 0.0001. **(b)** ATAC-seq analysis showing the cut counts in regions showing differential accessibility (>5 fold; P-value < 0.05) between MFD-1 and HET1A cells. Data are shown for MFD-1, OE33 and HET1A cells and grouped according to being more open in MFD-1 or HET1A cells. **(c)** UCSC genome browser tracks showing ATAC-seq cleavage data associated with the *BBOX1* (top) and *KRT8* (bottom) loci in HET1A, OE33 and MFD-1 cells. Regions of open chromatin associated with the TSS (arrows) are boxed. **(d)** *De novo* motif discovery of transcription factor binding sites over-represented in the regions that are either open in MFD-1 cells (top) or HET1A cells (bottom). **(e)** IPA GO term analysis of genes associated with a TSS showing changes (>5 fold) in open chromatin in MFD-1 compared to HET1A cells. The most highly significant terms associated with disease ontology are shown.

The overall conclusions of this Article are unaffected by the changes. The authors apologise for the errors.

