# Supplementary material for: Authentication and characterisation of a new oesophageal adenocarcinoma cell line: MFD-1
Source: Sci Rep. 2016 Sep 7;6:32417. doi: 10.1038/srep32417 (PMC5013399; doi:10.1038/srep32417)
Supplement: Supplementary Information [file srep32417-s1.doc]

Authentication and characterisation of a new oesophageal adenocarcinoma cell line: MFD-1

Edwin Garcia, Annette Hayden, Charles Birts, Edward Britton*, Andrew Cowie, Karen Pickard, Massimiliano Mellone, Clarisa Choh, Mathieu Derouet, Patrick Duriez, Fergus Noble, Michael J. White, John Primrose, Jonathan C. Strefford, Matthew Rose-Zerilli, Gareth J. Thomas, Andrew D. Sharrocks*, Rebecca C. Fitzgerald¥ & Timothy J. Underwood on behalf of the OCCAMS consortium.

Supplement Figure 1. Ploidy in MFD-1 cell line
